# Pulmonary arteriovenous malformation revealing Osler‐Weber‐Rendu disease: A case report

**DOI:** 10.1002/ccr3.5294

**Published:** 2022-01-17

**Authors:** Herveat Ramanandafy, Finaritra Princy Parfait Andriamahenina, Michel Harison Tiaray, Anjara Mihaja Nandimbiniaina, Angela Zamelina Razafindrasoa, Sonia Razafimpihanina, Diamondra Andriarimanga, Jean Noêl Solohery Ratsimbazafy, Jocelyn Robert Rakotomizao, Joëlson Lovaniaina Rakotoson, Hanta Marie Danielle Vololontiana

**Affiliations:** ^1^ Department of Internal Medicine University Hospital of Joseph Raseta Befelatanana Antananarivo Madagascar; ^2^ Department of Pneumology University Hospital of Joseph Raseta Befelatanana Antananarivo Madagascar

**Keywords:** arteriovenous malformations, Madagascar, Osler‐Weber‐Rendu disease, telangiectasias

## Abstract

Osler‐Weber‐Rendu disease is a genetic disease characterized by mucocutaneous and visceral telangiectasias. Pulmonary arteriovenous malformation is one of the main visceral complications revealing Osler‐Weber‐Rendu disease. The present case was a 34‐year‐old woman with exertional dyspnea and severe hypoxemia revealing pulmonary arteriovenous malformations on chest CT scan.

## Graphical abstract

The report of this case would allow the researchers in the domain of medicine to compare with the literature and could therefore constitute additional data on the diagnosis and management of Osler‐Weber‐Rendu disease.

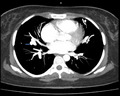

## 1 INTRODUCTION

Osler‐Weber‐Rendu disease or hereditary hemorrhagic telangiectasia is an age‐related autosomal dominant constitutional vascular dysplasia.^1^ The disease is rare and difficult to diagnose.^1^ The diagnosis is clinical, based on the presence of spontaneous and recurrent epistaxis, multiple telangiectasias, typical localization (lips, oral cavity, fingers, and nose), or visceral localizations, and a family history in the first generation.^1^ Its pathophysiology is linked to a perturbation of the balance between pro‐angiogenic factors, including the vascular endothelial growth factor, and anti‐angiogenic factors.^2^ It is also a serious disease due to its complications and its locations, especially in the brain. A pulmonary arteriovenous malformation is one of the major visceral complications that reveal the disease.^3^ We report the first Malagasy case of Osler‐Weber‐Rendu disease.

## 2 OBSERVATION

**FIGURE 1:**
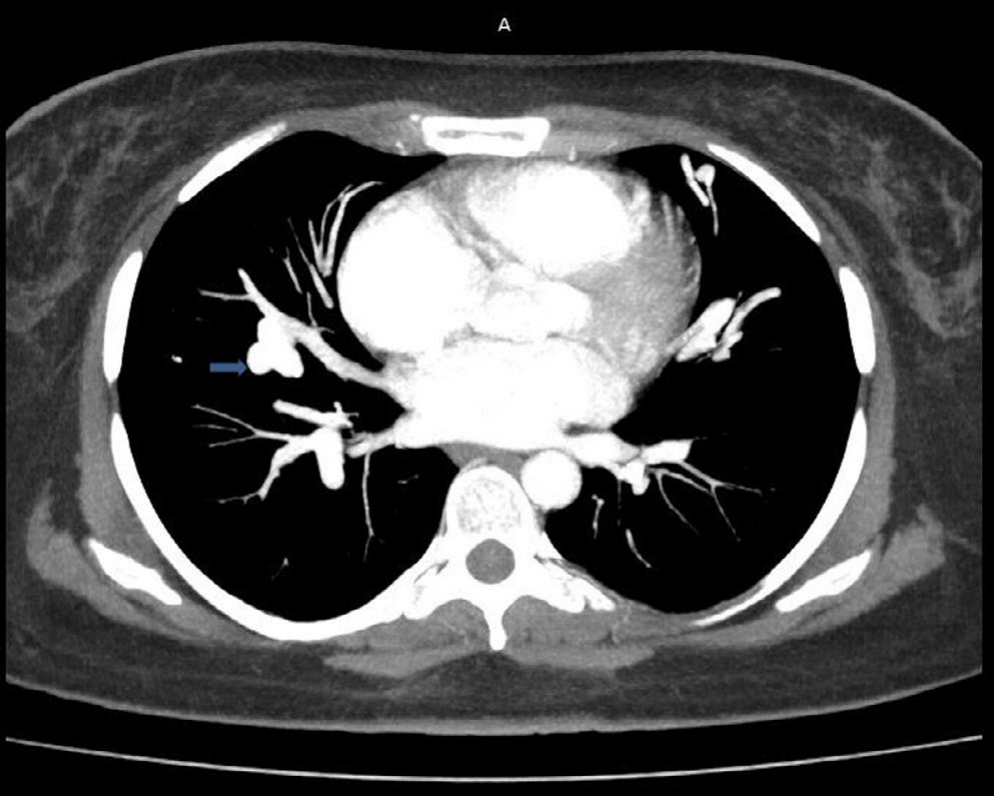
Mediastinal window chest CT scan with injection: pulmonary arteriovenous malformation of right pulmonary field

**FIGURE 2:**
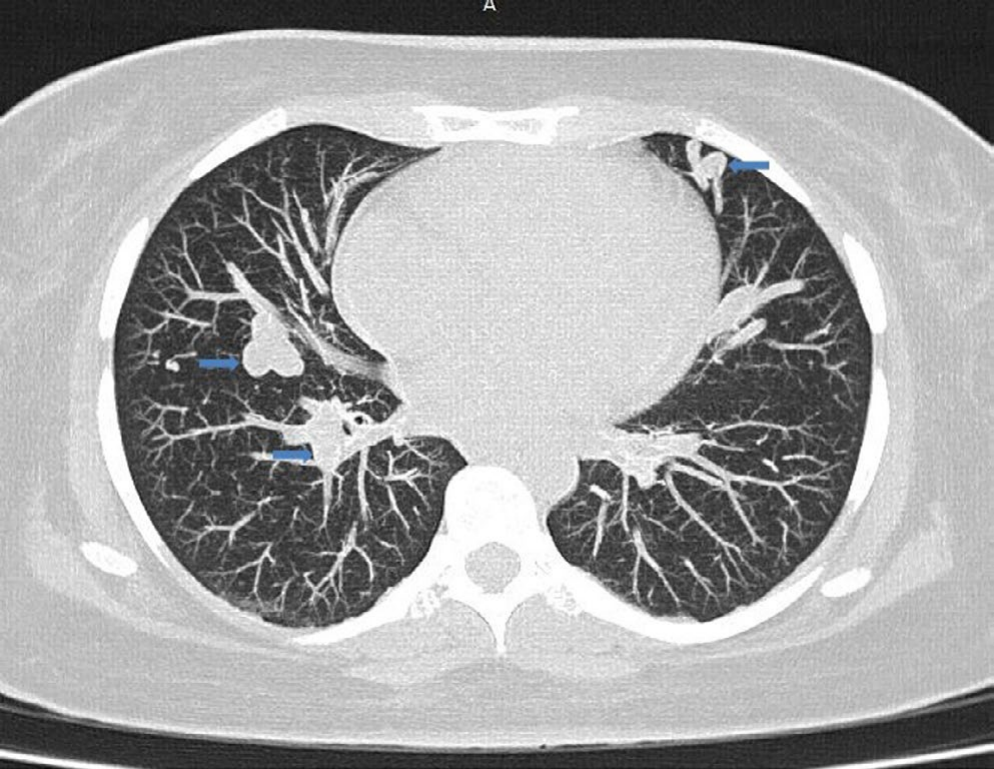
Parenchymal window chest CT scan with injection: multiple pulmonary arteriovenous malformations

It was a 36‐year‐old woman hospitalized in the Department of Pneumology, University Hospital of Joseph Raseta Befelatanana. The symptoms started 3 weeks ago with exertional dyspnea associated with intermittent coughs. The symptoms worsened despite a well‐managed treatment, which motivated her hospitalization in the department. In this history, episodes of dyspnea were reported in September 2020, recurrent epistaxis with family context in the first generation on the maternal side. The examination at admission found a polypneic patient at 22 cycles per minute with a drop in pulsed oxygen saturation (SpO2) to 83% in ambient air. The respiratory and cardiovascular examinations were unremarkable. There was no cyanosis, no digital hippocratism, and no telangiectasia. The otorhinolaryngeal examination objectified bilateral small drip epistaxis without functional repercussions. Blood cells count showed a hemoglobin level of 8.9 g/dl, an average blood volume of 62.5 fl, an average corpuscular concentration of 27.3 g/dl, and a platelet count of 377 G/L. Renal (serum creatinine = 42 μmol/L), hepatic functions (an aspartate aminotransferase = 17 U/L, an alanine aminotransferase = 21 IU/L), and coagulation profile (prothrombin time = 13.5 s, prothrombin level = 100%) were normal. Arterial gas measurements showed deep hypoxemia (PaO2 = 40.4 mm Hg) with severe hypocapnia (PCO2 = 22 mm Hg). The genetic analysis was not performed. Imaging such as thoracic computed tomography with injection of contrast product showed multiple peripheral and central vascular malformations corresponding to sacciform vascular dilations by arteriovenous fistula (Figures 1 and 2). Cardiac Doppler ultrasound revealed pulmonary arterial hypertension at 37 mm Hg with dilation of the right chambers (right ventricle = 45 mm, right atrium = 18 mm), flattening of the interventricular septum and minimal mitral insufficiency on Doppler examination. Contrast transthoracic cardiac ultrasound with intravenous injection of microbubbles could have helped to discover a right‐to‐left shunt, but is not available in Madagascar. The abdominal ultrasound was unremarkable; the liver was homogeneous of normal size without dilation of the hepatic veins. The diagnosis of Osler‐Weber‐Rendu disease was suggested by the presence of epistaxis, similar case in the family in the first generation and bilateral sacciform vascular dilations of the pulmonary fields on the chest CT scan. The patient was put on oxygen therapy, and for the epistaxis, the hemostatic procedures were prolonged bidigital compression and the initiation of oral tranexamic acid. About the pulmonary arteriovenous malformations, arterial embolization was proposed to improve the dyspnea, arterial gazometry, and also preventing complications. However, this technique was not performed due to the patient's condition. The outcome was good with oxygen therapy alone, which was maintained at 2 L/min.

## 3 DISCUSSION

Osler‐Weber‐Rendu disease was described by Rendu in 1896, the description was supplemented by Osler in 1902 and by Weber in 1907.^4^ The disease is due to a disturbance in the regulation of angiogenesis, linked to a genetic defect of autosomal dominant inheritance with variable expression.^2^ Penetrance is related to age and is complete around age of 40–50 years old.^5^ Three genes are associated with Osler‐Weber‐Rendu disease: ENG, ACVRL1, and MADH4 involved in the tumor growth factor signaling pathway. Mutation of only one of the two gene alleles is sufficient to cause the disease.^6^ This disease is rare, but ubiquitous. To the best of our knowledge, this was the first case reported in Madagascar. A few cases have been reported in the African literature.^7^ In France, the incidence of the disease was estimated at 1/6000 inhabitants.^8^ The diagnosis is based on the clinical criteria of Curaçao (Table 1).^1^ Recurrent epistaxis is the most common way of finding the disease. In the reported case, in front of marked respiratory signs with pseudoaneurysmal dilations of two pulmonary fields in chest CT scan, the pulmonary arteriovenous malformation was evoked. To sum up, our patient suffers from Osler‐Weber‐Rendu disease due to the presence of three Curaçao criteria, namely spontaneous epistaxis, reccurent epistaxis in the first generation family history and malformation arterioveinous pulmonary. In the literature, pulmonary arteriovenous malformation is one of the main visceral complications revealing Osler‐Weber‐Rendu disease, it is found in 15 to 33% of affected patients.^3^ Arteriovenous malformation is an abnormal communication between a pulmonary artery and a pulmonary vein, resulting in the creation of a low‐pressure right‐to‐left shunt, which can lead, depending on the flow of the shunt, to chronic hypoxemia. The clinical and biological signs are consequently dyspnea, cyanosis, digital hippocratism, and rarely polycythemia. Cyanosis and polycythemia may be masked by pre‐existing anemia associated with epistaxis. Dyspnea on exertion is the most common symptom (26%–86%); it is generally moderate and is thought to be correlated with the level of the shunt and the size of the pulmonary arteriovenous malformations.^3^ Chest radiography coupled with transthoracic cardiac contrast ultrasound with intravenous injection of microbubbles is excellent examinations for screening for arteriovenous malformation. Contrast transthoracic cardiac ultrasound can distinguish an intrapulmonary shunt from an intracardiac shunt and look for the presence of pulmonary arterial hypertension.^3^, ^9^ In the study by Cottin et al^10^, the combination of contrast ultrasound and chest X‐ray had a sensitivity of 100% and a specificity of 51% to diagnose a pulmonary arteriovenous malformation. If both examinations are positive, a chest CT scan is offered to confirm the diagnosis and specify the characteristics of the pulmonary arteriovenous malformation.^9^ Pulmonary arterioveinous hypertension is a rare complication of osler weber rendu disease. There are two types, either idiopathic most often linked to a mutation in the ACVRL1 ; or linked to a caediac hyperflow. It was diagnosed on a cardiac Doppler ultrasound in our case. In published reviews, cardiac ultrasound is the gold standard for screening and diagnosis of this complication.^3^ The treatment of pulmonary arteriovenous malformations is based on percutaneous arterial vaso‐occlusion (embolization), which improves dyspnea and prevents complications.^9^

**TABLE 1:** Diagnostic criteria according to the Curaçao consensus conference

1. Epistaxis	Spontaneous, recurrent.
2. Telangiectasias	Multiple: lips, oral cavity, fingers, and nose.
3. Visceral lesions	Pulmonary arteriovenous malformation, Gastrointestinal telangiectasias, Hepatic arteriovenous fistula, Cerebral and medullary arteriovenous malformation.
4. Family history	First generation related to a subject having the disease according to the previous criteria.

Note

The diagnosis of Osler‐Weber‐Rendu disease is as follows: defined if three criteria are present; Possible or suspected if two criteria are present; Probable if there are less than two criteria present.

## 4 CONCLUSION

Osler‐Weber‐Rendu disease is a rare genetic disorder, diagnosed by the clinical criteria of Curaçao and which should be confirmed by genetic testing. After epistaxis, the pulmonary arteriovenous malformation is one of discovery modes of the disease. Management of the disease must be multidisciplinary involving pulmonologists and vascular surgeons. Genetic counseling in relatives would be necessary for early detection of asymptomatic arteriovenous malformations.

## CONFLICT OF INTEREST

None declared.

## AUTHOR CONTRIBUTIONS

HR followed up the patient, collected the clinical data, and drafted the report. FPPA, AMN, AZR, JNSR, and DA followed up the patient. HMT, JNSR, MIR, and JRK designed and critically revised the report. JLR and HMDV validated the report. All the authors have read and approved the final draft of the manuscript. All authors contributed to this work and approved the final version.

## ETHICAL APPROVAL

The authors thank the patient who gave written permission for this report. The article does not contain any personal information that could identify the patient. The names and dates on the chest CT scan have been hidden. The authors have included only information necessary for scientific understanding.

## CONSENT

The development of this article has been approved by the patient and the department of pneumology who have given written permission for this work.

## Data Availability

The data that support the findings of this study are available from the corresponding author upon reasonable request.
